# Breaking Out of the Hive: A Case Report on Hypocomplementemic Urticarial Vasculitis Syndrome

**DOI:** 10.7759/cureus.60986

**Published:** 2024-05-24

**Authors:** Mena Louis, Adeel Akhtar, Brian Gibson

**Affiliations:** 1 General Surgery, Northeast Georgia Medical Center Gainesville, Gainesville, USA; 2 Internal Medicine, Northeast Georgia Medical Center Gainesville, Gainesville, USA; 3 Trauma and Acute Care Surgery, Northeast Georgia Medical Center Gainesville, Gainesville, USA

**Keywords:** personalized medicine, multidisciplinary approach, mycophenolate mofetil, rituximab, anca, hypocomplementemia, systemic vasculitis, leukocytoclastic vasculitis, huvs, hypocomplementemic urticarial vasculitis syndrome

## Abstract

Hypocomplementemic urticarial vasculitis syndrome (HUVS) is a rare autoimmune disorder characterized by chronic urticaria, systemic vasculitis, and hypocomplementemia, posing significant diagnostic challenges due to its overlap with common conditions and varied systemic manifestations. We report the case of a 36-year-old female with a history of post-birth cerebral hemorrhage and seizure disorder, who presented with abdominal pain, diarrhea, and subtle urticarial lesions. Initial investigations by gastroenterology suggested inflammatory bowel disease (IBD), but persistent symptoms and evolving cutaneous signs prompted further evaluation. A skin biopsy demonstrated leukocytoclastic vasculitis, while serological tests showed hypocomplementemia and positive antineutrophil cytoplasmic antibodies (ANCA), confirming HUVS. The patient's management included high-dose corticosteroids and mycophenolate mofetil, with partial symptom relief. Subsequent introduction of rituximab markedly improved her gastrointestinal and dermatological symptoms, highlighting its effectiveness in treating refractory HUVS. This case emphasizes the necessity for vigilance, interdisciplinary collaboration, and personalized treatment adaptations in managing HUVS.

## Introduction

Hypocomplementemic urticarial vasculitis syndrome (HUVS) is an uncommon autoimmune disease characterized by chronic urticaria accompanied by hypocomplementemia and systemic vasculitis [1]. This multifaceted disorder targets small vessels and presents with a spectrum of clinical manifestations, including skin lesions, joint pain, abdominal discomfort, and renal involvement [2].

Due to its systemic nature, HUVS can also affect multiple other organs, leading to potentially severe complications [3]. The syndrome is marked by low levels of circulating complement proteins and is often associated with autoantibodies, such as anti-C1q and antineutrophil cytoplasmic antibodies (ANCA), reflecting its complex immunopathological basis [4].

Diagnosing HUVS is challenging due to its overlapping symptoms with more common conditions such as chronic idiopathic urticaria and other forms of systemic vasculitis [5]. A definitive diagnosis typically hinges on the combination of clinical presentation, histopathological findings from skin biopsies, and specific laboratory markers of immune activation and complement consumption [6,7].

## Case presentation

A 36-year-old female with a significant medical history presented to the emergency department with concerns that initially centered around non-specific systemic symptoms. Her past medical history is notable for a cerebral hemorrhage occurring shortly after birth that necessitated the placement of a ventriculoperitoneal (VP) shunt and a resultant chronic seizure disorder managed with carbamazepine.

**Figure 1:**
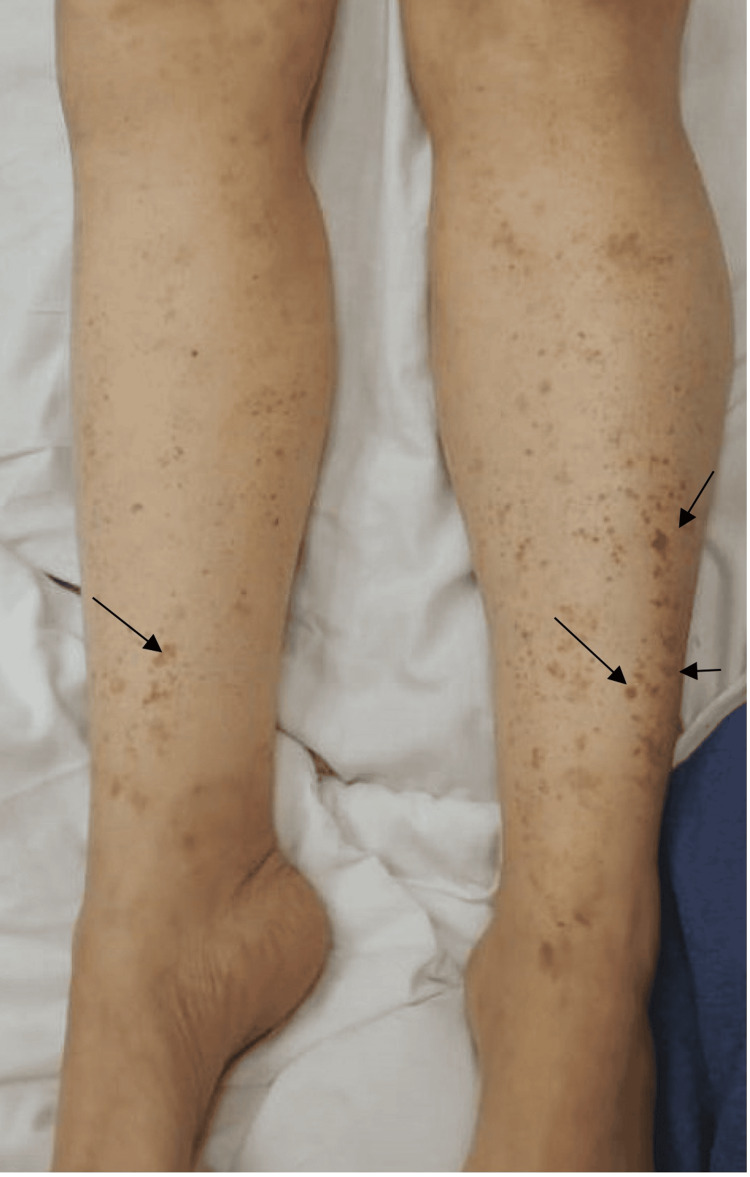
Bilateral lower extremities (arrows pointing to petechial rash)

The patient's current episode began with abdominal pain, diarrhea, and a petechial rash (Figure 1), accompanied by elevated inflammatory markers. The symptoms persisted over several months, with intermittent exacerbations. The petechial rash lasts over 24 hours and leaves behind hyperpigmentation and was initially subtle but became more pronounced over time.

Given the prominence of gastrointestinal symptoms, the initial diagnostic focus was directed toward potential inflammatory bowel disease (IBD) or intestinal angioedema. Computed tomography (CT) enterography was performed, revealing thickening of the small and large bowel walls, which further complicated the differential diagnosis. Extensive gastroenterological testing, including an esophagogastroduodenoscopy (EGD), colonoscopy, and capsule endoscopy, showed only scattered erythema without ulceration, failing to confirm IBD.

However, the evolving nature of the patient's skin lesions prompted dermatological and rheumatological consultations. A skin biopsy was essential in clarifying the diagnosis, revealing leukocytoclastic vasculitis consistent with hypocomplementemic urticarial vasculitis syndrome (HUVS).

Serological tests added complexity to her case; she tested positive for ANCA, with specificity for PR-3, and displayed hypocomplementemia. Additionally, an anti-C1q assay was elevated, further supporting the diagnosis of HUVS. All other baseline laboratory tests were unremarkable. These findings suggested a significant autoimmune component to her symptomatology, aligning with the HUVS diagnosis but also raising concerns for possible systemic vasculitis affecting the kidneys and other organs.

A kidney needle biopsy from the patient revealed a complex glomerulopathy with significant immune deposition. Notably, there was prominent deposition of C3 and lambda light chains, somewhat lesser amounts of C1q, IgG, and IgM, and only trace to mild IgA, with lambda staining more intense than kappa, including in vascular regions. The electron microscopy further identified "immune type" electron-dense deposits mainly in the mesangial areas, occasionally extending to subendothelial and subepithelial locations. These findings were accompanied by mesangial hypercellularity and proliferation, but no significant glomerulosclerosis was noted across approximately 40 glomeruli examined.

Pathological assessment indicates an immune-related glomerulopathy, potentially associated with the patient's clinical diagnosis of HUVS, although not presenting the typical full-house immunofluorescence pattern seen in lupus nephritis. This raises the possibility of a distinct or atypical form of immune complex disease. The presence of predominantly lambda light chains suggests a unique immune response or an underlying paraproteinemia, meriting ongoing surveillance. The biopsy also showed mild arteriosclerosis and tubular changes, but no direct evidence of vasculitis.

Management began with high-dose corticosteroids, initially with intravenous Solu-Medrol (methylprednisolone) at 30 mg every six hours, resulting in significant symptom improvement. After several days, treatment transitioned to oral prednisone at 60 mg daily with a planned taper. Despite partial relief, the patient remained dependent on high doses of steroids. Consequently, mycophenolate mofetil (CellCept) was added at 1,000 mg twice daily to further control systemic inflammation and vasculitis. However, her dependency on steroids remained high, prompting the consideration of more targeted therapies. After discussing potential risks and benefits, rituximab was initiated at a dose of 375 mg/m² weekly for four weeks to better control her immune response, given its efficacy in B-cell-mediated diseases. The patient responded positively after the second dose. Following this, her gastrointestinal and dermatological symptoms markedly improved. Post-treatment laboratory results showed normalized complement levels and decreased inflammatory markers, confirming the effectiveness of rituximab in managing refractory HUVS. Her musculoskeletal pain, however, persisted, requiring ongoing adjustments to her pain management approach. The patient was scheduled for regular follow-up to monitor the effectiveness of rituximab, assess for adverse effects, and adjust her treatment plan as needed.

## Discussion

Hypocomplementemic urticarial vasculitis syndrome is a rare and complex systemic autoimmune disorder characterized by long-lasting urticarial lesions, hypocomplementemia, and signs of systemic vasculitis [7]. The disease can affect multiple organ systems, including the skin, gastrointestinal tract, kidneys, and potentially the lungs and nervous system [8]. Given its wide range of manifestations, HUVS often presents significant diagnostic and therapeutic challenges [2].

The diagnosis of HUVS is particularly challenging due to its overlap with more common conditions such as chronic idiopathic urticaria, other forms of vasculitis, and autoimmune disorders such as systemic lupus erythematosus (SLE) [9]. Key to diagnosing HUVS is the presence of urticarial lesions that persist for more than 24 hours, often leaving behind hyperpigmentation, systemic symptoms, and low complement levels [10].

A biopsy of the affected tissue, usually the skin, showing leukocytoclastic vasculitis is crucial for confirmation [11]. Additionally, serological findings such as positive ANCA, anti-C1q antibodies, and hypocomplementemia help substantiate the diagnosis but can complicate differentiation from other autoimmune conditions. The biopsy was crucial as it provided direct histopathological evidence of vasculitis, distinguishing HUVS from other potential causes of her symptoms and guiding appropriate treatment. The biopsy is essential in such cases because it allows for the visualization of characteristic histopathological features, such as immune complex deposition and inflammatory cell infiltration, which are definitive for diagnosing vasculitis and ruling out other conditions. The complex presentation with multisystem involvement necessitated extensive investigations, underlining the importance of a comprehensive approach to diagnosis.

The management of HUVS requires a tailored approach, focusing on controlling the inflammatory process and addressing systemic involvement [12]. Corticosteroids, such as methylprednisolone, are commonly used for their potent anti-inflammatory effects [13]. However, long-term steroid use is associated with significant side effects, including osteoporosis, hyperglycemia, and increased infection risk [12].

For patients who exhibit partial response or require high doses of steroids, steroid-sparing agents such as mycophenolate mofetil (CellCept) or azathioprine may be employed. Additionally, rituximab, a monoclonal antibody against CD20 on B-cells, has been shown to be effective in cases resistant to traditional therapies [14]. Rituximab's role in B-cell depletion helps in reducing autoantibody production, which is crucial in autoimmune diseases such as HUVS [2].

The initiation of rituximab followed an extensive discussion of potential risks, including infection risks and hypogammaglobulinemia [2]. Her subsequent improvement shows the efficacy of rituximab in managing HUVS, although ongoing monitoring for adverse effects is imperative [13].

The differential diagnosis for this case included inflammatory bowel disease (IBD), intestinal angioedema, chronic idiopathic urticaria, systemic lupus erythematosus (SLE), and other forms of vasculitis. Initial evaluations, such as CT enterography, EGD, and capsule endoscopy, were directed toward ruling out IBD, which showed only scattered erythema without ulceration. Persistent symptoms and evolving cutaneous manifestations prompted further rheumatological and dermatological evaluations. Serological tests revealed hypocomplementemia, positive antineutrophil cytoplasmic antibodies (ANCA), and elevated anti-C1q, suggesting an autoimmune process. The final diagnosis of hypocomplementemic urticarial vasculitis syndrome (HUVS) was confirmed through a skin biopsy demonstrating leukocytoclastic vasculitis. Treatment options include high-dose corticosteroids, mycophenolate mofetil (CellCept), and rituximab. The patient received rituximab, which significantly improved her symptoms after two doses. Ongoing monitoring involves regular follow-ups and laboratory tests to assess treatment efficacy and side effects, such as osteoporosis, hyperglycemia, and increased infection risk from corticosteroids; gastrointestinal disturbances and bone marrow suppression from mycophenolate mofetil; and infusion reactions and infections from rituximab.

## Conclusions

This case exemplifies the intricate nature of diagnosing and managing hypocomplementemic urticarial vasculitis syndrome (HUVS), a condition characterized by its rarity and the complexity of its symptoms. The initial presentation of abdominal pain, non-specific gastrointestinal symptoms, and subtle urticarial lesions highlighted the challenges inherent in recognizing HUVS, which often mimics more common disorders such as inflammatory bowel disease and chronic idiopathic urticaria. The diagnosis was only clarified through persistent investigative efforts involving multiple specialties, emphasizing the necessity for a comprehensive and interdisciplinary approach. Skin biopsy findings of leukocytoclastic vasculitis, combined with serological markers such as hypocomplementemia and positive ANCA, were pivotal in confirming HUVS, illustrating the critical role of integrating clinical suspicion with histopathological and laboratory data to reach a definitive diagnosis.

In managing this case, the treatment strategy had to be tailored to address both the severity of the symptoms and the response to initial therapies. The use of high-dose corticosteroids provided initial relief but introduced concerns about long-term dependency and side effects, leading to the incorporation of mycophenolate mofetil and subsequently rituximab. The introduction of rituximab marked a significant turning point in her treatment, offering a reduction in steroid dependency and an improvement in quality of life through targeted B-cell depletion. This case highlights the importance of personalized medicine in the management of complex autoimmune disorders and reinforces the need for ongoing evaluation and adjustment of therapeutic strategies to optimize patient outcomes while minimizing adverse effects.
